# Ten-Year Molecular Surveillance of Drug-Resistant *Plasmodium* spp. Isolated From the China–Myanmar Border

**DOI:** 10.3389/fcimb.2021.733788

**Published:** 2021-09-01

**Authors:** Tongke Tang, Yanchun Xu, Long Cao, Penghai Tian, Jiang Shao, Yan Deng, Hongning Zhou, Bo Xiao

**Affiliations:** ^1^Institut Pasteur of Shanghai, Chinese Academy of Sciences, University of Chinese Academy of Sciences, Shanghai, China; ^2^School of Life Science and Technology, ShanghaiTech University, Shanghai, China; ^3^Yunnan Institute of Parasitic Diseases Control, Pu’er, China; ^4^CAS Key Laboratory of Molecular Virology and Immunology, Institut Pasteur of Shanghai, Chinese Academy of Sciences, Shanghai, China; ^5^Institutional Center for Shared Technologies and Facilities of Institut Pasteur of Shanghai, Chinese Academy of Sciences, Shanghai, China

**Keywords:** anti-malaria, drug-resistance, artemisinin, SNP mutation, China–Myanmar border

## Abstract

Antimalarial drug resistance has emerged as a major threat to global malaria control efforts, particularly in the Greater Mekong Subregion (GMS). In this study, we analyzed the polymorphism and prevalence of molecular markers associated with resistance to first-line antimalarial drugs, such as artemisinin, chloroquine, and pyrimethamine, using blood samples collected from malaria patients in the China–Myanmar border region of the GMS from 2008 to 2017, including 225 cases of *Plasmodium falciparum* and 194 cases of *Plasmodium vivax*. In artemisinin resistance, only the C580Y mutation with low frequency was detected in *pfk13*, and no highly frequent stable mutation was found in *pvk12*. In chloroquine resistance, the frequency of K76T mutation in *pfcrt* was always high, and the frequency of double mutations in *pvmdr1* of *P. vivax* has been steadily increasing every year. In pyrimidine resistance, *pfdhfr* and *pvdhfr* had relatively more complex mutant types associated with drug resistance sites, and the overall mutation rate was still high. Therefore, artemisinin-based combination therapies are still suitable for use as the first choice of antimalarial strategy in the China–Myanmar border region in the future.

## Introduction

Malaria is one of the major life-threatening infectious diseases in humans and is particularly prevalent in tropical and subtropical regions worldwide. Humans can be infected by five species of *Plasmodium*, including *Plasmodium falciparum*, *Plasmodium vivax*, *Plasmodium ovale*, *Plasmodium malariae*, and *P. knowlesi* (Singh and Daneshvar, 2013). *P. falciparum* and *P. vivax* have the highest incidences, followed by *P. ovale* and *P. malariae*, while some patients are also infected by *P. knowlesi*. Almost all malaria deaths occurring in Southeast Asia have been caused by *P. falciparum*. Globally, approximately 1.5 billion people were infected and 7.6 million patients died from malaria between 2000 and 2019. The majority of infections (82%) and deaths (94%) occurred in the WHO African Region, followed by the WHO South-East Asia Region (10% of infections, 3% of deaths) (WHO, 2020).

The effectiveness and longevity of antimalarial drugs in malaria control are largely dependent on in-depth studies of the determined markers *in vitro* and monitoring programs *in vivo*. In addition, it is critical to identify molecular markers that can predict resistance or reduce susceptibility to malaria to actively monitor the temporal trend of parasite susceptibility (Haldar et al., 2018). However, the emergence and spread of multidrug resistance have caused a permanent obstacle to the extinction of malaria. Malaria therapeutics have been plagued by recurring problems for more than 60 years. As first-line therapy, artemisinin-based combination therapies (ACTs) have been used for uncomplicated malaria infections in the Greater Mekong Subregion (GMS). However, the confirmed occurrence of artemisinin resistance in western Cambodia is a major threat to malaria control and elimination (Dondorp et al., 2009; Ashley et al., 2014). Both chloroquine (CQ) and sulfadoxine-pyrimethamine (SP) resistance have emerged in other areas of mainland Southeast Asia (Wellems and Plowe, 2001; Roper et al., 2004).

Mutations in the *pfk13* (artemisinin resistance-related gene) propeller domain are recommended for conducting molecular surveillance as an assistant tool for monitoring local resistance to artemisinin. At present, the mutations P441L, F446I, S449A, N458Y, P553L, V568G, P574L, and L675V in *pfk13* have been associated with delayed parasite clearance. In addition, the five mutations Y493H, I543T, R539T, R561H, and C580Y have been validated as artemisinin resistance mutations *in vitro* and *in vivo* (WHO, 2015) *Pvk12* was identified as a homolog of *pfk13* in *P. vivax* (Deng et al., 2016). Currently, there is no molecular evidence of high frequency mutations in *pvk12*, even in Southeast Asia (Deng et al., 2016; Deida et al., 2018; Tantiamornkul et al., 2018). The mutation V552I (Hassett et al., 2017) was subsequently observed in a *P. vivax* isolate from Cambodia and examined in this study.

The CQ resistance transporter gene of *P. falciparum* (*pfcrt*) is located on chromosome 7; it encodes a structural protein of the food vesicular membrane of *P. falciparum*, which functions as a CQ transporter. When the wild-type K76 of the carrier protein was mutated to threonine (T), the efficiency of CQ diffusion into the food vesicles of *Plasmodium* was decreased, and the *Plasmodium* showed resistance to CQ. Therefore, K76T can be used for clinical drug resistance monitoring of CQ (Nair et al., 2008; Dondorp et al., 2010; Lubell et al., 2011). The insertion of a lysine residue behind the tenth amino acid was observed in the homologous gene *pvcrt* of *P. vivax*, which may be related to CQ resistance (Basco and Ringwald, 1998); therefore, this site was also identified.

Field studies have shown that point mutations in *pfmdr1* in different geographical regions have different predictive values for CQ resistance and amino acid changes (Foote et al., 1990; Duraisingh et al., 1997). Five mutation sites in *Pfmdr1* (N86Y, Y184F, S1034C, N1042D, and D1246Y) have been reported to be associated with susceptibility to CQ (Antony and Parija, 2016). A polymorphism of *P. vivax pvmdr1*, which is homologous to that in *P. falciparum*, has been associated with CQ resistance in many studies (Brega et al., 2005; Villena et al., 2020). Whole-genome sequencing and microarray analysis of a single isolate of *P. vivax* from Pu’er revealed a selective pressure on putative drug resistance genes and found the previously reported T958M mutation to be associated with drug resistance (Orjuela-Sanchez et al., 2009; Dharia et al., 2010). The Y976F and F1076L mutations of *pvmdr1* are present in all malaria endemic areas where CQ is used as a first-line antimalarial drug (Suwanarusk et al., 2007; Kim et al., 2011; Lu et al., 2011; Rungsihirunrat et al., 2015). Isolates carrying only the F1076L mutation are not associated with CQ resistance (Barnadas et al., 2008; Orjuela-Sanchez et al., 2010). This observation supports the fact that resistance of *P. vivax* to CQ requires the presence of two mutations. F1076L is a prerequisite for the secondary product of Y976F, which is responsible for the reduction in CQ sensitivity (Barnadas et al., 2008). However, no other studies have found an association between Y976F mutations and resistance phenotypes (Fernandez-Becerra et al., 2009; Gama et al., 2009). Some studies have found an increase in the copy number of *pvmdr1*, which seems to be associated with increased susceptibility to CQ (Suwanarusk et al., 2007; Imwong et al., 2008; Lu et al., 2011; Melo et al., 2014). Recently, it has been shown that CQ resistance and clinical severity of *P. vivax* are associated with increased expression of *pvmdr1* and *pvcrt-o* (Sa et al., 2019), and the CQ resistance ortholog gene in *P. vivax* (also called *pvcrt* or *pvcg10*) (Sa et al., 2006).

The role of dihydrofolate reductase (*pfdhfr*) mutations in the mechanism of SP resistance has been well described (Wernsdorfer and Noedl, 2003). Pyrimidine resistance can be produced by a single point mutation (S108N) in *pfdhfr*, but the reductase reactivity to cyclochloroguanidine is only slightly reduced. A previous *in vitro* assay showed that the IC_50_ values of the single mutant (I164L) isolates were 6-fold higher than those of the wild-type and close to those usually reported for simple mutants S108N (roughly10-fold higher than wild type) (Andriantsoanirina et al., 2011). High resistance to pyrimidine requires coexisting mutations at other sites, including N51I or C59R. The mutation associated with cyclochloroguanidine resistance was S108N with A16V in *pfdhfr*. At the same time, the susceptibility of the resistant strain to pyrimidine did not vary significantly. Similarly, polymorphisms in *pvdhfr* and *pvdhps* are associated with antifolate drug resistance in *P. vivax*. The S58R and S117N polymorphisms in *pvdhfr* were highly correlated with pyrimidine resistance. Additional mutations, including P33L, N50I, F57L, T61M, V64L, and I173L, increased the degree of drug resistance and showed significantly high IC_50_ values for pyrimethamine (de Pecoulas et al., 1998; Hastings et al., 2004; Sinclair et al., 2011).

In this study, the polymorphism and prevalence of molecular markers associated with resistance to first-line antimalarial drugs, such as artemisinin, CQ, and pyrimethamine, were analyzed using blood samples collected from malaria patients in the China–Myanmar border region of the GMS over a 10-year duration. The results of this study will help develop novel treatment strategies for malaria patients with antimalarial drug resistance.

## Materials and Methods

### Ethics Statement

These studies were approved by the Yunnan Institute of Parasitic Diseases (No.: 202009) and performed according to the Ethical Committee guidelines. Genetic testing was performed on stored blood samples obtained as part of routine diagnostic work-up for patients with symptoms of malaria. Although there is an absence of risk and anonymous data processing, informed consent was obtained for the collection of samples from persons suspected of malaria.

### Subjects and Malarial Blood Sample Collection

From 2008 to 2017, a total of 419 venous blood samples on filter paper, including 225 cases of *P. falciparum* malaria and 194 cases of *P. vivax* malaria, were smeared and observed under an optical microscope to confirm the presence of the corresponding type of pathogen. No other clinical symptom was recorded. The samples were collected from malaria parasite-infected patients in the China–Myanmar border region of the GMS. Subjects who agreed to participate in the study took blood from their fingertips and dropped it on a filter paper with a diameter of approximately 5 mm. After drying in air, the samples were placed in a sealed plastic bag and stored at 4°C for a long time. All the samples were provided by the Yunnan Institute of Parasitic Diseases.

### Preparation of DNA Template From Blood Samples

Blood membranes with a diameter of 5 mm were taken from each sample, and the Blood DNA Kit (Omega D3392-02) was used for *Plasmodium* genome extraction. The procedure was modified slightly based on the manufacturer’s instructions: samples were heated with proteinase K at 56°C for 1 h to improve the extraction rate. Finally, 30 µl of polymerase chain reaction (PCR)-grade H_2_O was used to elute the DNA into the QSP EP tube. The DNA template was then concentrated to 10 µl at 45°C using a Thermo concentrator. The concentrated DNA sample (3 µl) was used as the PCR template, and the remaining DNA samples were stored in a refrigerator at −20°C.

### Amplification of Drug-Resistance Gene

Nested PCR amplification using 2×Taq PCR Mix (Tiangen, KT201) was performed to amplify the resistance genes in each DNA sample from patients infected with *P. falciparum* (the kelch domain of *pfk13*, *pfcrt*, *pfmdr1*, *and pfdhfr*) or *P. vivax* (*pvk12*, *pvcrt-o*, *pvmdr1*, *and pvdhfr*). The primer sequences and reaction product fragment sizes for the two rounds of PCR are listed in **Supplementary Table S1**. The total volume for the first round of the nested PCR reaction was 10 µl containing 3 μl DNA template, 5 μl 2×Taq PCR mix, and 1 μl each of Primer-PF and Primer-PR (10 mmol/L). The first round PCR conditions were as follows: denaturation at 94°C for 3 min; 35 cycles of denaturation at 94°C for 30 s, annealing at 50°C for 30 s, and extension at 62°C 3 min; and final extension at 62°C for 7 min. The total volume of the second round PCR was 50 µl containing 2 μl amplified product from the first round as the DNA template for the second round of PCR, 25 μl 2×Taq mix, 2 μl each of Primer-NF and Primer-NR (10 mmol/L), and then sterile ddH_2_O to make up the reaction system to 50 μl. The conditions of the second round of PCR were denaturation at 94°C for 3 min; 35 cycles of denaturation at 94°C for 30 s, annealing at 50°C for 30 s, and extension at 62°C for 60 s; and final extension at 62°C for 7 min. One microliter of the second-round amplification products and positive amplification products was used for electrophoresis detection using 1% and 2% agarose gels, respectively. Positive bands were selected, and DNA was extracted using a Gel Extraction Kit (Omega D2500-02). DNA marker was purchased from Sangon Biotech (Shanghai, China). DNA fragments were sequenced by Parsons Biotech (Shanghai, China).

### SNP Statistics and Sequence Analysis

Sequencing results, together with resistance-related gene sequences of *the P. falciparum* wild-type standard strain 3D7 (ID No. for *pfk13*: PF3D7_1343700, *pfcrt*: PF3D7_0709000, *pfmdr1*: PF3D7_0523000, *pfdhfr*: PF3D7_0417200), *P. vivax* strain P01 (ID no. for *pvk12*: PVP01_1211100), and *P. vivax* standard strain (*pvcrt*:PVX_087980, *pvmdr1*: PVX_080100 *pvdhfr*: PVX_089950) were aligned on the PlasmoDB website, using MEGA5.04 software to translate the DNA sequences into corresponding amino acid sequences, count SNPs, and analyze correlations with their drug resistance sites (Matlani et al., 2021).

## Results

### The Geographical Origin of the Study Samples

**Figure 1:**
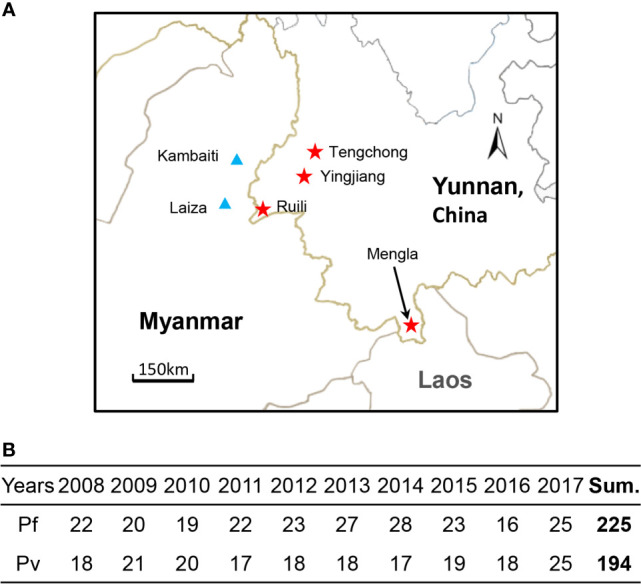
Information about blood samples. **(A)** Geographic locations of blood samples. The red star represents the region of Yunnan, China, and the blue triangles represent the region of Myanmar. The map was produced using Photoshop. **(B)** The number of *P. falciparum*- and *P. vivax*-infected malaria patient samples in 10 years from 2008 to 2017.

Blood samples were collected from Gambati and Laiza in Kachin State of Myanmar and Tengchong, Yingjiang, Ruili, and Mengla in the Yunnan Province of China (**Figure 1A**). A total of 419 blood samples, including 225 cases of *P. falciparum* and 194 cases of *P. vivax (* **Figure 1B**), were collected during the 10-year period from 2008 to 2017. The blood sample was taken from the fingertips, placed on a 5-mm filter paper, dried in air, and stored in a sealed plastic bag at −20°C for a prolonged duration.

### Artemisinin Resistance-Related Genes, *Pfk13* and *Pvk12*

**Figure 2:**
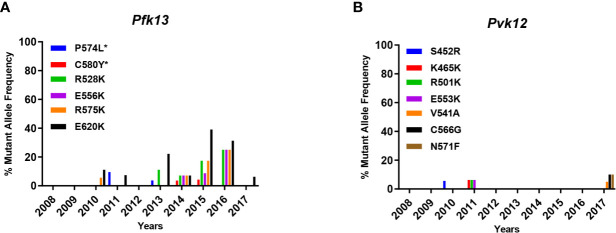
Mutant locus frequency and distribution of *pfk13* and *pvk12*. **(A, B)** Histogram analysis for *pfk13* and *pvk12* in **Table 1**, respectively. The star indicates the loci associated with artemisinin resistance.

The *P. vivax* K12 gene, which is homologous to *pfk13* in *P. falciparum*, was studied based on the mutation sites of molecular markers of artemisinin-associated resistance genes. As shown in **Table 1**, one mutant C580Y was identified in *P. falciparum* in 2014 and 2015. In 2016 and 2017, this point mutation did not appear and it was speculated to have disappeared; however, there were still chances of unknown vulnerabilities. Mutation P574L was reported in 2011 and 2013. This study also included some mutations at a higher frequency, such as R528K, E556K, R575K, and E620K, whose mutant frequencies were greater than 5%, and it can be intuitively observed that there was an increasing trend from 2013 to 2016 (**Figure 2A**). It is reasonable to assume that these point mutants were associated with the development of artemisinin resistance, but they should be specifically validated in insect strains *in vitro*. There was no locus of V552I as reported in *P. vivax*, but the presence of mutation codons S452R, K465K, R501K, and E553K was observed, which then disappeared in 2010 and 2011. Until 2017, V541A, C566G, and N57I showed slightly higher mutation frequencies of 5%, 10%, and 10%, respectively, in the collected samples. Further studies are needed to verify whether these point mutations lead to artemisinin resistance. In general, there were no stable high-frequency mutant loci in *P. vivax* samples (**Figure 2B**).

**Table 1:** Statistical analysis of the mutation sites and their frequency in *pfk13* and *pvk12*.

	*P. falciparum* (*Pfk13*)		*P. vivax* (*Pfk12*)	
Years	Amino acid and genetic changes^a^	Proportion (%)	Amino acid and genetic changes^a^	Proportion (%)
		(Mutants/n)		(Mutants/n)
2008	ND (0/22)	ND (0/22)	ND (0/18)	ND (0/18)
2009	ND (0/20)	ND (0/20)	ND (0/21)	ND (0/21)
2010	R575K(A**G**A→A**A**A)	5.6 (1/18)	S452R^c^(**A**GC→**C**GC)	5.6 (1/18)
	E620K^b^(**G**AA→**A**AA)	11.1 (2/18)		
2011	P574L (C**C**T→C**T**T)	4.8 (1/21)	K465K^b^ (AA**G**→AA**A**)	6.25 (1/16)
	E620K^b^(**G**AA→**A**AA)	9.5 (2/21)	R501K^b^ (A**G**A→A**A**A)	6.25 (1/16)
			E553K^b^ (**G**AA→**A**AA)	6.25 (1/16)
2012	ND (0/23)	ND (0/23)	ND (0/18)	ND (0/18)
2013	P574L(C**C**T→C**T**T)	3.7 (1/27)	ND (0/18)	ND (0/18)
	R528K^b^ (A**G**A→A**A**A)	11.1 (3/27)		
	E620K^b^ (**G**AA→**A**AA)	22.2 (6/27)		
2014	C580Y (T**G**T→T**A**T)	3.6 (1/28)	ND (0/17)	ND (0/17)
	R528K^b^ (A**G**A→A**A**A)	7.1 (2/28)		
	E556K^b^ (**G**AA→**A**AA)	7.1 (2/28)		
	E620K^b^ (**G**AA→**A**AA)	7.1 (2/28)		
	R575K (A**G**A→A**A**A)	7.1 (2/28)		
2015	C580Y (T**G**T→T**A**T)	4.3 (1/23)	ND (0/19)	ND (0/19)
	R528K^b^ (A**G**A→A**A**A)	17.4 (4/23)		
	E556K^b^ (**G**AA→**A**AA)	8.7 (2/23)		
	R575K (A**G**A→A**A**A)	17.4 (4/23)		
	E620K^b^(**G**AA→**A**AA)	39.1 (9/23)		
2016	Y493F (T**A**C→A**A**C)	6.25 (1/16)	ND (0/18)	ND (0/18)
	R528K^b^ (A**G**A→A**A**A)	25 (4/16)		
	E556K^b^ (**G**AA→**A**AA)	25 (4/16)		
	R575K (A**G**A→A**A**A)	25 (4/16)		
	E620K^b^(**G**AA→**A**AA)	31.25 (5/16)		
2017	E620K^b^(**G**AA→**A**AA)	8 (2/25)	V541A^b^(GTG→GCG)	5 (1/20)
			C566G^b^(**T**GT→**G**GT)	10 (2/20)
			N571F^b^(**AA**C→**TT**C)	10 (2/20)

a Mutation sites detected, the mutant amino acid in bold.

b None reported mutation spots detected in China–Myanmar border before 2020; n, the total number of samples under year; ND no mutations were detected in this study.

### CQ Resistance-Related Genes, *Pfcrt*, *Pvcrt-o*, *Pfmdr1*, and *Pvmdr1*

**Figure 3:**
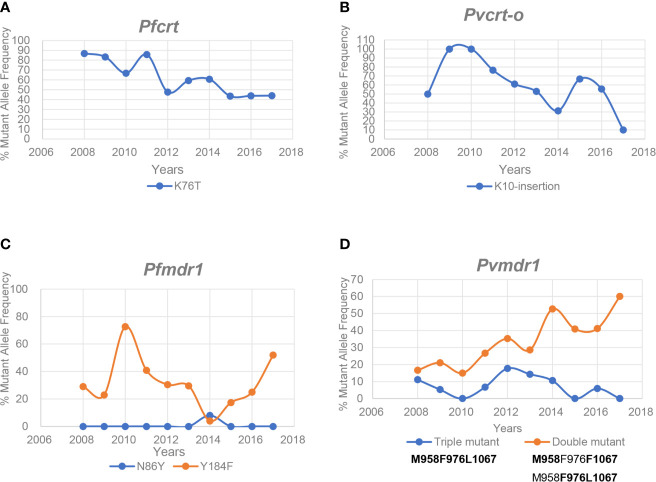
Genotype frequency variation of *pfcrt*, *pvcrt-o*, *pfmdr1*, and *pvmdr1*. **(A, B)** Mutant locus frequency of K76T in *pfcrt* and K10 insertion in *pvcrt-o*. **(C)** Mutant locus frequency of N86Y in *pfmdr1* is given in blue and red, respectively. **(D)** Mutant locus frequency of triple mutant and double mutant in *pvmdr1* is given in blue and red, respectively.

K76T is the CQ-resistant marker of *pfcrt* in *P. falciparum*, and K10 insertion is the CQ-resistant marker of *pvcrt-o* in *P. vivax*. These markers showed a general decreasing trend in mutant frequency over the 10-year period (**Figures 3A, B**). However, K76T in *pfcrt* still had a high mutation frequency (>40%) in 2017 (**Figure 3A**), which may have led to the poor therapeutic effect of CQ against *P. falciparum* malaria. As the frequency of K10 insertion in *pvcrt-o* decreased to 10% in 2017 (**Figure 3B**), reusing CQ may be considered for *P. vivax* malaria treatment in this region.

Another factor affecting CQ resistance is the polymorphism of *Pfmdr1*. Only N86Y and Y184F of *pfmdr1* were detected in *P. falciparum* samples, of which only one N86Y mutant locus was found in 2014. The frequency of Y184F locus dropped to 4% in 2014, but subsequently reached 52% in 2017 (**Figure 3C**). Overall, the main factor for CQ resistance in this region was the widespread prevalence of K76T in *pfcrt*. In the samples of *P. vivax*, three mutations were detected in *pvmdr1*, including Y976F, F1067L, and T958M; there may be simultaneous mutations in each mutant strain, possibly yielding multiple combinations. T958M is a secondary product of mutation formation and was observed in all mutant strains. The combination type of **M958F976L1067** showed the highest resistance rate to CQ. Fortunately, its frequency remained low. However, the double mutant frequency of **M958F976**F1067 and **M958**Y976**L1067** showed a steady increasing trend with each passing year, reaching 60% in 2017 (**Figure 3D**).

### Pyrimidine Resistance-Related Genes, *Pfdhfr* and *Pvdhfr*

**Figure 4:**
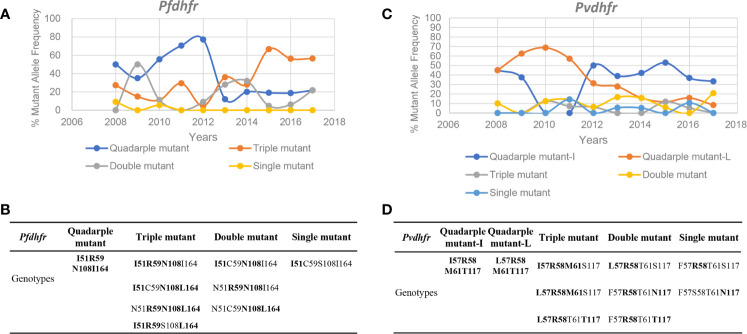
Genotype frequency variation of *pfdhfr* and *pvdhfr*.** (A, C)** Popular tendency of all genotypes from 2008 to 2017 in *pfdhfr* and *pvdhfr* according to the list as given in **(B, D)**, respectively.

N51I, C59R, S108N, and I164L were detected in *pfdhfr* of *P. falciparum*, and there were several combinations in the same mutant strain (**Figures 4A, B**). The four-fold mutant form **I51R59N108I164** was highly resistant to pyrimethamine, followed by the triple mutant form containing S108N. Quadruple mutations were dominant and maintained a high mutant frequency until 2012, which began to decrease sharply from 77.3% in 2012 and remained at approximately 20% in the next few years (**Figure 4A**). The frequency of triple mutations increased to 56.5% in 2017, which may indicate that resistance to pyrimethamine was reduced. F57I/L, S58R, T61M, and S117N/T were detected in *pvdhfr* of *P. vivax*, along with multiple mutant types (**Figures 4C, D**), in which multiple mutant forms containing S58R or S117N were highly resistant to pyrimethamine. *pvdhfr* has always been dominated by quadruple mutations, but the polymorphism F57I/L may have changed from F57L to F57I in 2011, which may be related to the control of medication.

## Discussion

In this study, we successfully performed a molecular surveillance of the mutation loci of the genes responsible for resistance to several first-line antimalarial drugs, such as artemisinin, CQ, and pyrimethamine, by analyzing their resistance-related gene polymorphisms in samples collected from the China–Myanmar border region from 2008 to 2017. Overall, for both *P. falciparum* and *P. vivax*, mutations of artemisinin resistance genes are becoming more and more complex and diverse, but the CQ and pyrimethamine drug resistance marker gene mutation rate has declined. For artemisinin resistance, only C580Y at low frequency was detected in *pfk13*, and no stable mutation with high frequency was found in *pvk12*. For CQ resistance, K76T in *pfcrt* has shown an overall declining trend since 2008, while keeping a high mutation frequency. The frequency of K10 insertion in *pvcrt-o* of *P. vivax* was almost 100% in 2008 and 2009, and decreased gradually each year, to about 10% in recent years. These indicated the unreliability of this marker for CQ resistance in *P. vivax*. As the CQ resistance in *P. vivax* has been linked to *pvcrt* (*pvcrt-o*) transcription level, a genetic-cross study supported an upregulated *pvcrt* expression as a mechanism of CQ resistance (Sa et al., 2019); this was also supported by a recent field study (Rovira-Vallbona et al., 2021). Herein, we observed that the frequency of double mutants in *pvmdr1* has been steadily increasing each year. In general, CQ may not be suitable as a first-line treatment for a long time in this region. For pyrimidine resistance, there were complex types of genotypes in drug resistance-related loci in both *pfdhfr* and *pvdhfr*, and the overall mutation frequency was high. In summary, artemisinin is still available as a first-line drug in this region.

In terms of artemisinin resistance, our study not only clarified the mutation history of *pfk13* in the China–Myanmar border region over the past ten years, but also detected some PfK13 amino acid non-synonymous mutation trends. In *pfk13*, in addition to the locus C580Y, which has been confirmed to be related to drug resistance in previous reports, four other loci were detected: the non-validated mutation locus R575K, which has been reported with a high mutation rate along the Thai-Myanmar border as early as 2007 and with low mutation frequency in other GMS regions (Nyunt et al., 2015; Talundzic et al., 2015), and the three mutant loci, R528K (Laminou et al., 2018), E556K (Guerra et al., 2017), and E620K (de Laurent et al., 2018), which have been reported with a low mutant frequency in the Africa regions recently, but with a high steady mutant frequency in the China–Myanmar border in this study. Some mutation sites (P574L and C580Y) detected in this study were similar to those reported by other studies conducted in Myanmar or the China–Myanmar border region using parasites collected from the China–Myanmar border region from 2007 to 2012 (Wang et al., 2015) and to those detected in Myanmar and relevant border regions of Thailand and Bangladesh between 2013 and 2014 (Tun et al., 2015). The F446I mutation is the most prevalent mutation at the China–Myanmar border and north of Myanmar, and introduction of F446I mutation into PfK13 leads to increased ring survival rates in *P. falciparum* isolates *in vitro*, but no mutation was detected in our study (Wang et al., 2018). These results indicated that the artemisinin resistance marker *Pfk13* mutations are very complex and exhibited an increasing trend. Our study provides a strong theoretical basis for subsequent ACTs, but new mutant loci that are directly related to developing resistance by *in vitro* parasites require to be studied further using allelic exchange genetic modification methods, such as CRISPR/Cas9 genomic editing, combined with *in vitro* drug screening tests (Xiao et al., 2019; Huang et al., 2020). The mechanism of action of artemisinin remains controversial, and our study provides some evidence of artemisinin drug resistance. *In vivo* artemisinin resistance has been proposed (Noedl, 2005) and identified by the presence of significantly decreased parasite reduction rates, manifested clinically by markedly longer parasite clearance times from the body (Noedl et al., 2008; Noedl et al., 2010). A recent study suggested that interconnected mechanisms might poise K13 mutant parasites to survive dihydroartemisinin treatment and increase the proteostatic capacity of the parasite. These mechanisms include an enhanced ability to eliminate damaged proteins through the unfolded protein response and ubiquitin–proteasomal machinery, and remodeling of secretory and vesicular transport processes that impact hemoglobin endocytosis and protein and lipid trafficking (Mok et al., 2021).

However, there are still some limitations in this study. We only collected about 20 samples of *P. falciparum* and *P. vivax* from 2007 to 2018 in the China–Myanmar border, and thus, the expansion of the sample size to make a more representative conclusion and further experimental validations to the new mutation pattern of the artemisinin resistance gene *PfK13* are urgently needed. Overall, the new mutations reported in our study may provide a basis for further research on novel molecular mechanisms of antimalarial drugs as well as on malaria patient treatment.

## Data Availability Statement

The original contributions presented in the study are included in the article/**Supplementary Material**. Further inquiries can be directed to the corresponding authors.

## Ethics Statement

This study was approved by the Yunnan Institute of Parasitic Diseases and according to Ethical Committee. Genetic testing was performed on stored blood samples obtained as part of routine diagnostic work-up patients with symptoms of malaria. Despite the absence of risk and the anonymous data processing, the informed consent form has been signed when collecting samples of suspected malaria patients.

## Author Contributions

TT, YX, PT, and JS performed the experiments. TT, LC, and BX participated in data analysis. TT, LC, and BX wrote and revised the manuscript. YD and HZ provided the samples and revised the manuscript. TT and BX conceived the study and participated in the study design. All authors contributed to the article and approved the submitted version.

## Funding

This research was supported by the National Natural Science Foundation of China (31900428), the Key Collaborative Research Program of the Alliance of International Science Organizations (ANSO-CR-KP-2020-06), China Postdoctoral Science Foundation (2019M651597), Shanghai Post-doctoral Excellence Program (2019260), Special Research Assistant Project of Chinese Academy of Sciences, Pu’er Municipal Expert Workstation of L. J., and the innovation Capacity Building Project of Jiangsu province (BM2020019).

## Conflict of Interest

The authors declare that the research was conducted in the absence of any commercial or financial relationships that could be construed as a potential conflict of interest.

## Publisher’s Note

All claims expressed in this article are solely those of the authors and do not necessarily represent those of their affiliated organizations, or those of the publisher, the editors and the reviewers. Any product that may be evaluated in this article, or claim that may be made by its manufacturer, is not guaranteed or endorsed by the publisher.
